# Everolimus for pediatric patients with acute graft-versus-host disease after hematopoietic stem cell transplantation: A pilot study

**DOI:** 10.1097/MD.0000000000008464

**Published:** 2017-11-03

**Authors:** Yu-Hua Chao, Yin-Chen Chang, Han-Ping Wu, Ching-Tien Peng, Te-Fu Weng, Kang-Hsi Wu

**Affiliations:** a Department of Pediatrics, Chung Shan Medical University Hospital; b School of Medicine, Chung Shan Medical University; c Department of Nursing, College of Medicine and Nursing, Hungkuang University; d Division of Pediatric General Medicine, Department of Pediatrics, Chang Gung Memorial Hospital; e College of Medicine, Chang Gung University, Taoyuan; f Division of Pediatric Hematology-Oncology, Children's Hospital, China Medical University; g Department of Biotechnology and Bioinformatics, Asia University; h School of Post-Baccalaureate Chinese Medicine, College of Chinese Medicine, China Medical University, Taichung, Taiwan.

**Keywords:** acute graft-versus-host disease, everolimus, hematopoietic stem cell transplantation

## Abstract

Acute graft-versus-host disease (aGVHD) is a significant cause of morbidity and mortality after allogeneic hematopoietic stem cell transplantation (HSCT). Due to the poor prognosis for patients not responding to first-line steroids treatment, improvements in aGVHD therapy are needed. Everolimus is a promising candidate that combines immunosuppressive properties with anti-neoplastic effects. Here, we retrospectively reviewed the efficacy of everolimus with steroids as primary treatment in 13 patients with grade II to grade IV aGVHD after HSCT. Among them, 12 (92.3%) had complete response to everolimus with steroids without additional immunosuppressive agents. The median duration of therapy was 76 days (range 20–110). Asymptomatic hypertriglyceridemia was the most common therapy complication (69.2%), but treatment interruption was not needed. Thrombotic microangiopathy was rare (7.7%), but can be quickly solved by stopping everolimus and cyclosporine treatment. Other toxicities were manageable. Two patients developed chronic GVHD (15.4%), limited in one and extensive in the other. The overall survival was 76.9% with a median follow-up of 3.4 years after HSCT (range 0.7–5.7). Accordingly, everolimus with steroids were feasible for patients with aGVHD after HSCT as primary treatment. Further large-scale studies are required.

## 1 Introduction

Allogeneic hematopoietic stem cell transplantation (HSCT) has become a standard of curative approach for a vast of diseases. Graft-versus-host disease (GVHD) is a significant cause of morbidity and mortality after allogeneic HSCT, and continues to be a major limitation to successful HSCT. Steroids are considered standard primary therapy for acute GVHD (aGVHD). The response rate to initial therapy was approximately 25% to 50% in adults^[1–3]^ and 76% in pediatric patients.^[4,5]^ The prognosis is dismal for those not responding to first-line treatment. Therefore, aGVHD treatment needs further improvement.

Everolimus, a new-generation mammalian target of rapamycin inhibitors, combines immunosuppressive properties with anti-neoplastic and anti-viral effects. Everolimus has demonstrated efficacy in the prevention of allograft rejection after solid organ transplantation,^[6–9]^ and in the prophylaxis of GVHD^[10]^ and treatment of chronic GVHD after HSCT.^[11–13]^ Comparing with traditional immunosuppressive agents, the researchers found that the major benefits of everolimus lie in the lack of nephrotoxicity and in its anti-neoplastic effects, demonstrating the potential of everolimus in the management of aGVHD after HSCT.

To our knowledge, no report regarding the use of everolimus for aGVHD after allogeneic HSCT has been published in the literature. Herein, we share useful experience in our institution.

## 2 Patients and methods

### 2.1 Patients

This study was approved by the institutional review board of the China Medical University & Hospital (CMUH105-REC2-145). The clinical records of patients who underwent allogeneic HSCT between August 2011 and July 2016 in the Children's Hospital of China Medical University Children's Hospital were retrospectively reviewed. Thirteen consecutive patients who developed grade II to grade IV aGVHD and received everolimus with steroids as primary treatment were enrolled for analysis.

### 2.2 Conditioning regimens

Regimens of conditioning depended on the underlying disease. Patients with acute myeloid leukemia received busulfan (1 mg/kg every 6 h for 4 days) and cyclophosphamide (60 mg/kg/d for 2 days) as conditioning. For acute lymphoblastic leukemia and chronic myeloid leukemia, the conditioning regimen included cyclophosphamide (60 mg/kg/d for 2 days) and total body irradiation (2 Gy twice a day for 3 days). Patients with myelodysplastic syndrome received busulfan (1 mg/kg every 6 h for 4 days), cyclophosphamide (60 mg/kg/d for 2 days), and melphalan (140 mg/m^2^/d for 1 day). The conditioning regimen for patients with severe aplastic anemia was cyclophosphamide (60 mg/kg/d for 2 days) and fludarabine (25 mg/m^2^/d for 5 days). Patients with Diamond–Blackfan anemia were treated with busulfan (1 mg/kg every 6 h for 4 days) and cyclophosphamide (50 mg/kg/d for 4 days) as conditioning.

### 2.3 GVHD prophylaxis

Patients received cyclosporine and methotrexate for GVHD prophylaxis, with the adjunction of rabbit anti-thymocyte globulin (2.5 mg/kg/d for 4 days) in those receiving a graft from an unrelated or mismatched donor. Patients with the stem cell source from cord blood received GVHD prophylaxis with cyclosporine, methylprednisolone, and rabbit anti-thymocyte globulin. The target trough levels of cyclosporine were between 150 and 300 ng/mL.

### 2.4 Treatment for aGVHD

The diagnosis and organ-specific grading of aGVHD were based on Glucksberg modified consensus criteria.^[14,15]^ During the study interval between August 2011 and July 2016, all patients with grade II to grade IV aGVHD after HSCT received everolimus with steroids as primary treatment in our institution. Everolimus was administered orally starting on the day of aGVHD onset, with an initial dose of 1.6 mg/m^2^/d in 2 divided doses. Serum concentrations were measured twice a week, and the dose of everolimus was subsequently adjusted to achieve target trough levels of 3 to 8 ng/mL. Methylprednisolone at 2 mg/kg/d was given concurrently.

### 2.5 Supportive care and assessment of side effects

Supportive care was performed according to our institutional guidelines. Prophylactic antibiotics consisted of ciprofloxacin, micafugin, trimethoprim-sulfamethoxazole, and acyclovir. Patients were evaluated periodically for adverse events, organ functions, and changes in aGVHD. Laboratory evaluation, including a complete blood count and chemistry panel, hepatic and renal function tests, and urine analysis, was performed twice a week. Both cytomegalovirus (CMV) pp65 antigen and quantitative polymerase chain reaction were tested every week to detect CMV reactivation. In the event of a positive test result, ganciclovir was administered intravenously. Granulocyte colony-stimulating factor was given until absolute neutrophil counts were greater than 1.0 × 10^9^/L.

### 2.6 Definition of response

Complete response was defined as resolution of aGVHD sustained for at least 1 month with no additional treatment required. Partial response was defined as the improvement of aGVHD grade in at least 1 evaluated organ without deterioration in other organs. Patients who had persistent aGVHD requiring additional immunosuppressive therapies were considered unresponsive to the primary therapy of everolimus and steroids.

## 3 Results

From August 2011 to July 2016, a total of 28 patients received allogeneic HSCT in our institution. Eighteen patients developed aGVHD, and the rate of aGVHD was 64.3%. Six patients had chronic GVHD. The overall survival rate was 71.4% with follow-up periods ranging from 7 months to 5.7 years. A total of 13 patients had grade II to grade IV aGVHD, and all of them received everolimus with steroids as primary treatment. The clinical characteristics of the 13 patients are summarized in Tables 1 and 2. All patients were less than 20 years old, and the median age at HSCT was 15.0 years (range 1.1–19.0). Majority of patients (10 of 13) received allogeneic HSCT for hematologic malignant disease. Except for 1 patient with chronic myeloid leukemia blast crisis, these patients were in complete remission at the time of HSCT. Two patients were allografted for severe aplastic anemia, and 1 for Diamond–Blackfan anemia. The hematopoietic stem cell sources were peripheral stem cells in most patients (11 of 13). Five patients received a graft from a sibling donor, including 1 (Patient 11) with a 4/6 matched donor. Seven patients were grafted from a matched unrelated donor, and 1 (Patient 3) received cord blood transplantation from a 4 of 6 mismatched donor.

**Table 1:**
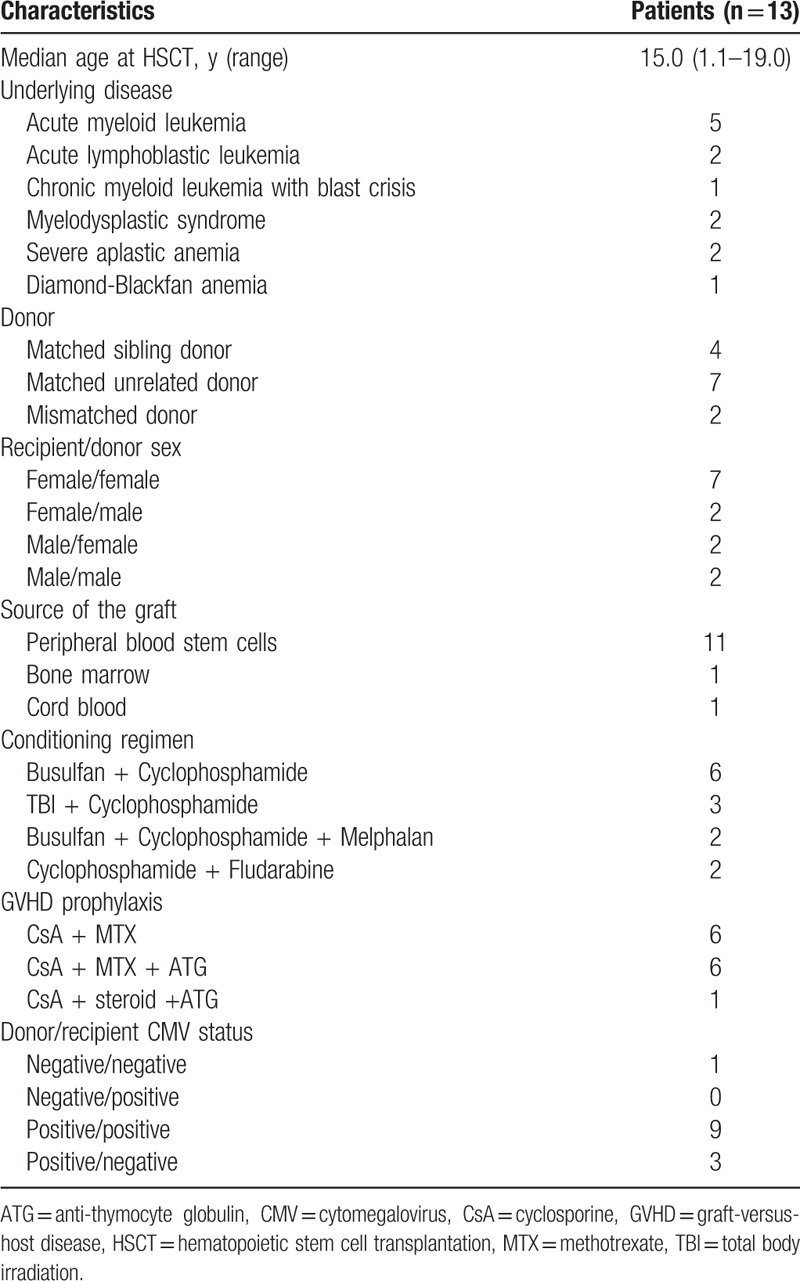
Baseline characteristics.

**Table 2:**
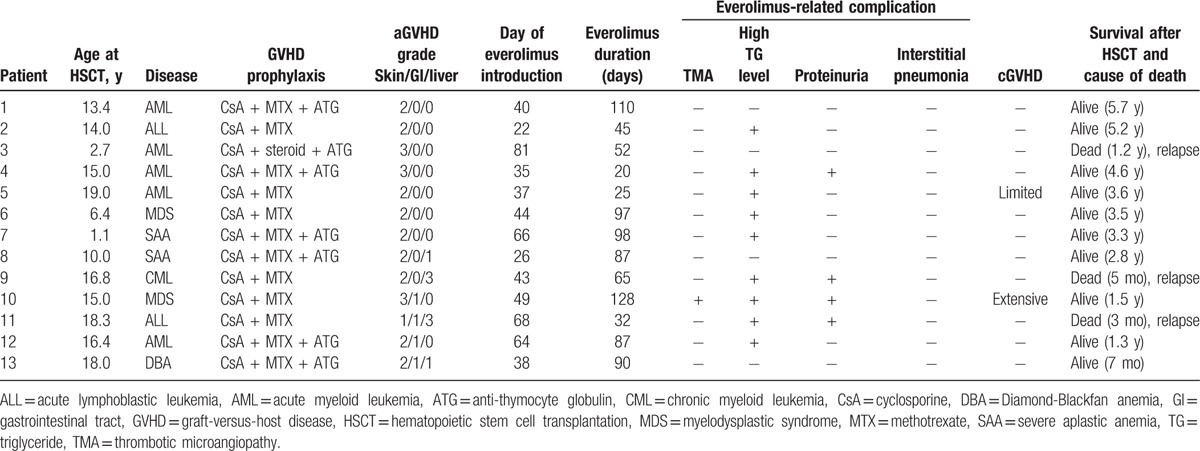
Summary of individual patient outcomes.

The skin was the most frequent site of aGVHD (all patients). The liver was the target of aGVHD in 4 patients, and 2 of them had grade III hepatic involvement. Everolimus with steroids was given as primary treatment for aGVHD in all 13 patients, and the median day of everolimus introduction was 43 after HSCT (range 22–81 days). Except for 1 patient (Patient 10), all had complete response to treatment without additional immunosuppressive agents (92.3%, 12 of 13). None had flares of aGVHD, and the median duration of therapy was 76 days (range 20–110).

Asymptomatic hypertriglyceridemia was the most common complication of therapy (69.2%, 9 of 13), but did not require treatment interruption. Statin therapy was effective in reducing serum triglyceride levels, and no cardiovascular events were observed in these patients. Proteinuria was observed in 4 patients (30.8%), but did not require additional medication and modification of therapy. Decreased platelet counts with abnormally elevated schistocyte levels, compatible with the criteria for thrombotic microangiopathy (TMA),^[16]^ was noted in 1 patient (Patient 10) at 4 months after the initiation of therapy. Everolimus was replaced by mycophenolate mofetil for aGVHD control, and cyclosporine was discontinued. TMA quickly resolved. At 2 months following the discontinuation of everolimus, he developed CNS aspergillosis and prolonged use of steroids may be an important contributing factor. No interstitial pneumonia was recorded. Ten patients (76.9%) had CMV reactivation and were effectively treated with ganciclovir. No severe bacterial infection was observed during the treatment periods for aGVHD. Two patients developed chronic GVHD later, limited in Patient 5 and extensive in Patient 10. Ten patients were alive with a median follow-up of 3.4 years after HSCT (range 0.7–5.7). Three patients died due to a relapse in their underlying disease.

## 4 Discussion

aGVHD remains a major limitation to successful allogeneic HSCT, and steroids constitute the present standard primary therapy. In the previous 5-year period before August 2011, the rate of aGVHD was 45.7% (16 of 35 allogeneic HSCT recipients) in our institution and methylprednisolone at 2 mg/kg/d was the first-line therapy for those with grade II to grade IV disease. Compatible with the previous reports,^[1–5]^ the response rate was only 60%. The prognosis was dismal for those not responding to the primary therapy. As the efficacy of sirolimus in aGVHD^[17–21]^ and chronic GVHD,^[22,23]^ everolimus, which is a hydroxyethylester derivative of sirolimus, was added to the primary regimen for grade II to grade IV aGVHD in our institution. Twelve of the 13 patients (92.3%) in this setting had complete response to the combination of everolimus and steroids without significant adverse events. The response rate was much better than that of steroids alone.

A higher incidence of sinusoidal obstruction syndrome and TMA associated with the use of everolimus and tacrolimus for GVHD prophylaxis was reported.^[10]^ However, none of the patients in the present study developed sinusoidal obstruction syndrome with the use of everolimus for their aGVHD after HSCT. TMA was rare (7.7%) in our study, compatible with the observations on HSCT recipients with chronic GVHD.^[11,12]^ The most common complication probably associated with everolimus in the present study was hypertriglyceridemia (69.2%), and these patients were asymptomatic without treatment interruption. Other side effects were manageable. As the long-term use of steroids is associated with a well-characterized burden of complications, this approach may allow for a faster tapering of steroids in patients with aGVHD.

Although our study population was small and limited in pediatric patients, our data showed that everolimus with steroids appeared to be an effective and well-tolerated first-line treatment option for patients with aGVHD following HSCT. With the combination of steroids in the present study, evaluating the individual role of everolimus in the treatment of aGVHD is difficult. However, our results provided a rationale for further prospective studies that use everolimus in aGVHD after HSCT as primary therapy.
